# Maternal and perinatal factors associated with childbirth-related post-traumatic stress symptoms: a cross-sectional study

**DOI:** 10.3389/fpsyg.2026.1779037

**Published:** 2026-07-13

**Authors:** Lucyna Wójcicka, Anna Weronika Szablewska, Arkadiusz Prajzner, Agata Zdun-Ryżewska, Agnieszka Czerwińska-Osipiak, Dagmara Klasa-Mazurkiewicz

**Affiliations:** 1 Division of Obstetric and Gynaecological Nursing, Faculty of Health Sciences with the Institute of Maritime and Tropical Medicine, Medical University of Gdańsk, Gdańsk, Poland; 2 Institute of Psychology, Faculty of Pedagogy and Psychology, University of the National Education Commission, Kraków, Poland; 3 Division of Quality of Life Research, Department of Psychology, Faculty of Health Sciences with the Institute of Maritime and Tropical Medicine, Medical University of Gdańsk, Gdańsk, Poland; 4 Division of Gynaecology and Obstetrics, Department of Gynaecology, Obstetrics and Neonatology, Medical University of Gdańsk, Gdańsk, Poland

**Keywords:** birth trauma, caesarean section, obstetric complications, perinatal mental health, postpartum period, post-traumatic, stress disorders

## Abstract

**Objective:**

The aim of this study was to examine how a set of selected interacting maternal and perinatal clinical characteristics are associated with symptoms of post-traumatic stress after birth.

**Methods:**

We conducted a cross-sectional online survey among women between 3 and 18 months after birth. Participants completed self-report measures of post-traumatic stress symptoms and questions regarding clinical variables related to pregnancy, birth and the postnatal period. Statistical models were used to estimate the association between clinical factors and symptom severity.

**Results:**

The strongest predictors of elevated post-traumatic stress symptoms were short-term complications like postpartum curettage and the need for hospitalisation due to neonatal health complications. Emergency caesarean section during the first stage of labour was also associated with higher symptom severity, whereas planned caesarean section, postpartum haemorrhage and prolonged postnatal stay were not significantly related to symptoms.

**Conclusion:**

These findings highlight the importance of recognizing acute obstetric and neonatal events as relevant factors contributing to post-traumatic stress after birth. They specify that trauma-informed perinatal care must be actively implemented by establishing targeted psychological screening protocols for women experiencing emergency surgical interventions or neonatal separations, and by prioritizing transparent communication and continuous consent during acute obstetric procedures.

## 1 Introduction

### 1.1 Prevalence and consequences of childbirth-related trauma

Childbirth is a profound physiological and psychological event that, although typically anticipated as a positive life transition, may also be experienced as traumatic. Recent evidence indicates that while 4–6% of mothers meet the full diagnostic criteria for post-traumatic stress disorder (PTSD) after birth, and up to 12% report clinically significant symptoms (Heyne et al., 2022), the estimated prevalence of clinical childbirth-related PTSD (CB-PTSD) in Central and Eastern Europe, including Poland, is 3.4%, with reported rates ranging from 1.8 to 6.1% (Murawska et al., 2026).

The risk is particularly elevated after emergency caesarean section, with prevalence ranging from 2% to over 40% depending on clinical circumstances (Orovou et al., 2025). The disorder extends beyond the individual, affecting maternal–infant bonding, breastfeeding duration and child emotional development, and contributes to elevated parenting stress and the risk of depression (Ayers et al., 2018; Dikmen-Yildiz et al., 2017; Garthus-Niegel et al., 2018). These outcomes highlight that CB-PTSD is not merely a personal psychological burden but a public-health issue with intergenerational consequences. This problem takes on particular significance in the Polish context, which is characterised by one of the highest caesarean section rates in Europe, reaching 44.4% (Euro-Peristat, 2022), and according to recent reports, even exceeding 50% in some centres (Zahorowska et al., 2025). In the face of this distinct and growing trend toward the high medicalisation of childbirth, investigating the link between acute obstetric interventions and childbirth-related trauma becomes a matter of fundamental importance for public health in Poland.

Postpartum post-traumatic stress disorder (PP-PTSD) is a broad term encompassing any post-traumatic stress symptoms occurring in the postpartum period. However, when the traumatic stressor is directly related to the labour experience, the condition is more precisely operationalized as childbirth-related post-traumatic stress disorder (CB-PTSD). Although neither PP-PTSD nor CB-PTSD appear as distinct diagnosis in classification systems like DSM-5 and ICD-11. The present study focuses on trauma and delivery experiences. Therefore, for conceptual clarity, the term CB-PTSD will be used consistently throughout this paper to denote post-traumatic stress symptoms specific to childbirth. The present study also adopts the DSM-5 framework to birth-related trauma. According to the Diagnostic and Statistical Manual of Mental Disorders, Fifth Edition (DSM-5) criteria, which require exposure to an event involving actual or threatened death, serious injury or threat to one’s physical integrity (American Psychiatric Association, 2022). In the context of childbirth, this core cluster (A) the exposure is directly linked to the experience of labour and delivery. According to DSM-5 diagnosis requires the presence of symptoms from four core clusters for more than 1 month: (B) recurring nightmares, flashbacks, constituting intrusive symptoms; (C) avoiding stimuli and situations similar to the traumatic event; (D) persistent fear, negative beliefs, and other negative changes in mood and alterations in cognitions; and (E) exaggerated startle response, hypervigilance and/or arousal and reactivity. These symptoms must cause clinically significant distress or functional impairment.

### 1.2 Conceptual models and risk factors

Evidence from systematic reviews consistently shows that the ethology of PTSD after birth is multifactorial, involving pre-existing vulnerabilities, perinatal complications, subjective perceptions of birth and social support systems (Khsim et al., 2022; Orovou et al., 2025). Based on clinical observations, it might be inferred that traumatic birth experiences are connected to emergency caesarean section, instrumental delivery, obstetric complications, and perceived loss of control or lack of emotional support during labour (Carter et al., 2022; Hüner et al., 2024). Neonatal complications, admission to intensive care and mother-infant separation have also been identified as risk factors for perinatal PTSD (Di Chiara et al., 2022; Malouf et al., 2024; Orovou et al., 2023). Although the term PP-PTSD refers to the timing of symptoms in the postpartum period, in the vast majority of cases, perinatal trauma is the cause, making childbirth-related post-traumatic stress disorder (CB-PTSD) the primary underlying factor in postpartum distress (Policy Center for Maternal Mental Health, 2025). Despite this progress, research has been predominantly focused on Western European and high-income populations, with far fewer studies exploring how clinical and psychosocial factors intersect in Central and Eastern European contexts (Ďuríčeková et al., 2024; Murawska et al., 2026; Riklikienė et al., 2025).

Several important gaps remain in the literature. First of all, in most studies, risk factors are assessed in isolation-examining single obstetric events rather than recognizing that these procedures and complications often occur simultaneously or sequentially during childbirth. This approach limits the understanding of their cumulative burden and relative impact on psychological trauma. We must accept the possibility that acute clinical medical events may be interrelated, creating a distress cascade that cannot be captured by bivariate analyses. Therefore, to assess how the broad spectrum of maternal and perinatal characteristics collectively shapes trauma responses, it is necessary to employ multivariate statistical models that can control for confounding effects and estimate the unique predictive value of each clinical factor. Childbirth-related trauma is commonly conceptualised within a biopsychosocial framework (Engel, 1977, 1980). Our broader research programme has examined different dimensions of this framework across several studies, including psychological and contextual determinants of childbirth experiences (Brewin and Burgess, 2014; Giannandrea et al., 2013; Horsch et al., 2024; Mackle et al., 2023). The present study was designed to focus specifically on the contribution of biological and clinical perinatal factors to CB-PTSD symptoms. Therefore, the analyses reported here primarily address the biological-clinical domain and its association with psychological outcomes. Although the present study did not include dedicated measures of social support or broader socioeconomic determinants, it incorporated several care-related variables reflecting interpersonal and contextual aspects of maternity care. Consequently, while the primary focus of the analyses was on biological-clinical factors and psychological outcomes, selected elements of the care environment were also considered.

Infant-related clinical characteristics are included in few analyses, despite neonatal health outcomes and early separation from the mother may strongly influence trauma responses. Finally, there is a shortage of data from countries with distinct maternity-care systems and varying levels of access to perinatal psychological support. Addressing these limitations is necessary to refine current conceptual models of CB-PTSD and ensure their applicability across diverse healthcare contexts.

### 1.3 The polish context

In Poland, perinatal mental health remains a relatively underexplored area. Despite the introduction of National Perinatal Care Standards aimed at ensuring safe and person-centred maternity care (Regulation of the Minister of Health of August 16, 2018, on the Organizational Standard of Perinatal Care (Journal of Laws 2018, Item 1756, as Amended), 2018) both service-user reports and professional assessments indicate persistent gaps in communication, continuity and psychological support during childbirth (Fundacja Rodzić po Ludzku, 2021). These challenges coexist with one of the highest caesarean section rates in Europe (Amyx et al., 2024), and limited availability of postpartum mental-health screening. The absence of validated instruments tailored to childbirth-related trauma has further constrained research and early identification of affected women. This shortfall is particularly relevant in the context of Poland’s declining birth rate and increasing public concern over reproductive health and experiences of maternity care (Trepczyk and Szablewska, 2024).

### 1.4 Current study

The present cross-sectional study was conducted among postpartum women in Poland to examine how maternal perinatal and neonatal clinical characteristics (as reported by mothers) collectively relate to symptoms of post-traumatic stress after birth. To justify our analytical approach, this study attempts to answer three research questions:

1. How do various acute clinical events, modeled simultaneously, predict the severity of CB-PTSD symptoms?
2. What is the contribution of specific mode of delivery compared to near-term postpartum interventions and neonatal complications?
3. Which associated clinical factors significantly increase the odds of a woman developing probable CB-PTSD?

By simultaneously examining these factors in an integrated approach, the study aims to elucidate their unique and relative contribution to CB-PTSD and to provide context-specific evidence that may inform trauma-informed perinatal care practices and guide policy development within both national and broader European frameworks. This multifactorial predicting design provides the direct foundation for our statistical modelling strategy, moving from isolated associations toward a comprehensive approach to risk modelling.

## 2 Materials and methods

### 2.1 Study design and participants

This study employed a cross-sectional observational design aimed at examining the relationship between maternal and perinatal clinical characteristics and the severity of post-traumatic stress symptoms after childbirth. The research formed part of a broader project previously approved by the Bioethics Committee of the Medical University of Gdańsk (KB/540/2024) and conducted in accordance with the 1964 Declaration of Helsinki. The reporting of this study followed the Strengthening the Reporting of Observational Studies in Epidemiology (STROBE) guidelines (von Elm et al., 2014).

Data were collected between 12 and 31 December 2024 through an anonymous online survey. Participants were recruited through popular, local and nationwide Polish parenting forums and social media groups (ranging from 3,000 to over 100,000 active members), including the Facebook groups for, e.g., “Rodze w 2024,” “Ciąża i Poród - wsparcie dla mam,” “Mama i dziecko - ciąża, macierzyństwo,” “Ciąża i macierzyństwo po 30, 40,” “Rodzę w Krakowie – grupa dla przyszłych mam,” “Rodzę w Pułtusku,” “Hallo gdzie rodzić - Warszawa?” or specific sub-forums on parenting portals like BabyBoom.pl. To reduce the possibility of selection bias, sensitivity was given to exclude support groups for traumatized mothers or PTSD patients. Moreover, the invitation was issued neutrally, by asking mothers to describe their childbirth and postpartum period in general terms, rather than focusing on complicated or traumatic experiences.

The initial screening included 303 submissions from respondents. Eligibility criteria included maternal age ≥ 18 years, proficiency in Polish and having an infant aged 3–18 months. Women within 2 months postpartum or reporting severe psychiatric, cognitive or chronic medical conditions were excluded. This selected group was intended to exclude women at high risk of developing psychological complications in the postpartum period, based on participants reported previous treatment history and naturally occurring mood disorders in the early postpartum period. It was assumed that removing this major source of confounding would enable testing of the relationships considered in this study. The informed consent procedure required participants to review an electronic information sheet describing study objectives, procedures and confidentiality measures, followed by active consent confirmation prior to accessing the questionnaire. To improve the quality of the study sample, the data collection form was designed to automatically reject incomplete responses and responses from individuals who did not consent to participate in the study.

**Figure 1:**
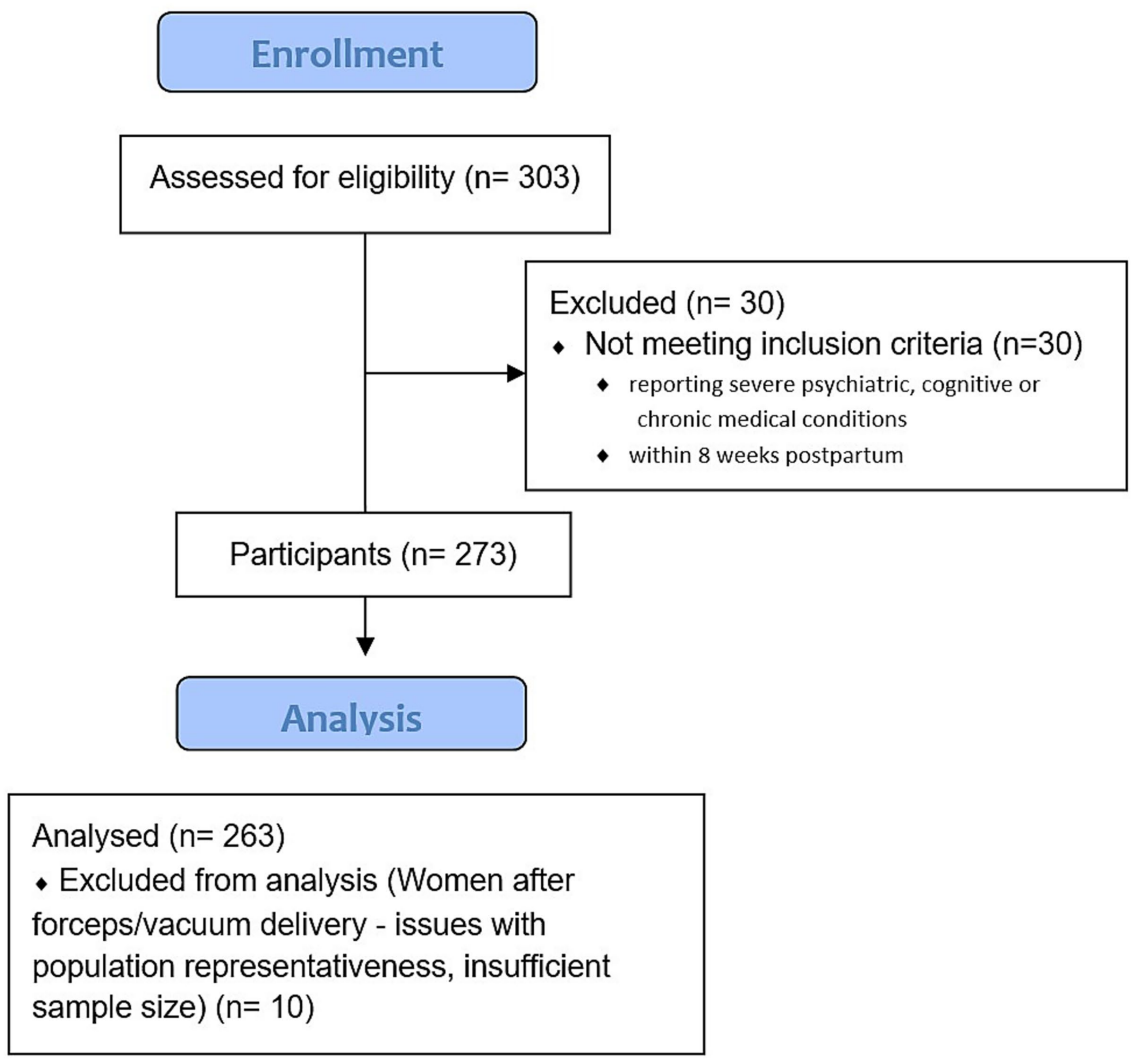
Participant flow diagram.

The analytic sample was reduced to 263 postpartum women by excluding participants who did not meet the inclusion criteria and all expedited birth modes (vacuum- or forceps-assisted births). Deliveries involving forceps or vacuum assistance were specifically excluded from the analysis due to concerns regarding population representativeness and insufficient sample size. A graphical representation of participant recruitment for analysis is presented in Figure 1.

### 2.2 Measures

#### 2.2.1 Sociodemographic, obstetric and clinical variables

Participants completed a structured questionnaire assessing sociodemographic data (e.g., age, education, parity, socioeconomic status), obstetric and perinatal history (e.g., pregnancy complications, mode and stage of delivery, gestational age, postpartum procedures), and infant outcomes (e.g., congenital anomalies, hospitalisation, medical complications). Clinical variables were grouped into composite categories and transformed into dichotomous values for statistical analysis. Specifically, composite variables were operationalized as follows: ‘intrapartum complications’ related to the need to use obstetric manoeuvres, e.g., during shoulder dystocia; ‘near-term complications’ included postpartum haemorrhage and postpartum curettage; ‘further-term complications’ like extensive perineal trauma, complications of wound healing/infection or post-puncture syndrome; ‘medicalisation of labour’ encompassed procedures such as episiotomy, induction/stimulation with oxytocin, pre-induction of labour, amniotomy, or labour analgesia in general (with the use of drugs and anaesthetics) and other procedures like need for medication, catheterization, etc.; ‘facilities during delivery’ involved aspects like free vocalization during labour, continuous consent, free choice of position during childbirth, early skin-to-skin contact with the newborn, possibility of eating during labour, childbirth with a companion and breastfeeding within 2 h of birth; and ‘neonatal-related hospitalisation’ was defined by admission to Level II or III Neonatal Care, hyperbilirubinemia /phototherapy, perinatal infection/antibiotic therapy, asphyxia in the perinatal period or respiratory distress. To recognize a complex feature as present, it was sufficient for the participant to indicate at least one of the associated detailed factors.

The survey was designed by a multidisciplinary team (midwives, obstetricians, psychologists, and biostatisticians) to ensure clarity and accessibility for participants recruited online. Female participants ranged in age from 21 to 42 years (*M* = 30.33, SD = 4.07). Detailed characteristics of demographic and grouped clinical factors were presented in Table 1.

**Table 1:** Study group characteristic.

Tested variable			*n*	%
Demographic	Education	Primary	4	1.52
		Secondary	50	19.01
		Higher	209	79.47
	Place of residence	Rural area	30	11.41
		City up to 50,000 citizens	43	16.35
		City 50–500,000 citizens	52	19.77
		City over 500,000 citizens	138	52.47
	Relationship	Single	4	1.52
		Non-formal	51	19.39
		Formal	208	79.09
Clinical	Mode of birth	Vaginal birth	137	52.09
		CS planned	46	17.49
		CS I stage	55	20.91
		CS II stage	25	9.51
	Intrapartum complications: The need to use obstetric manoeuvres, e.g., during shoulder dystocia	Absence	241	91.63
		Occurrence	22	8.37
	Near-term complications: Postpartum haemorrhage	Absence	244	92.78
		Occurrence	19	7.22
	Near-term complications: Postpartum curettage	Absence	240	91.25
		Occurrence	23	8.75
	Further-term complications: Extensive perineal trauma Complications of wound healing/infection Post-puncture syndrome	Absence	251	95.44
		Occurrence	12	4.56
	Medicalisation of labour: Episiotomy Induction/stimulation of labour with oxytocin Amniotomy Pre-induction of labour Labour analgesia Other procedures: need for medication, catheterization, etc.	Absence	60	22.81
		Occurrence	203	77.19
	Prolonged hospital stay (over 5 days)	Absence	199	75.67
		Occurrence	64	24.33
	Facilities during delivery: Ongoing consent Possibility of free vocalization Possibility of active childbirth and choice of position Possibility of eating during labour Childbirth with a companion Skin-to-skin contact with the newborn for 2 h after birth Breastfeeding within 2 h of birth	Absence	44	16.73
		Occurrence	219	83.27
	Duration of last pregnancy	Premature	54	20.53
		Term gestation	209	79.47
	Child congenital defect	Absence	255	96.96
		Occurrence	8	3.04
	Neonatal-related hospitalisation: (The newborn’s condition required extended neonatal care beyond routine physiological monitoring) Admission to Level II Neonatal Care Admission to Level III Neonatal Care Hyperbilirubinemia/phototherapy Perinatal infection/antibiotic therapy Asphyxia in the perinatal period Neonatal respiratory distress	Absence	160	60.84
		Occurrence	103	39.16

#### 2.2.2 Assessment of post-traumatic stress symptoms

Symptoms of childbirth-related, post-traumatic stress were evaluated using two validated Polish-language self-report instruments:

- The Postpartum PTSD Questionnaire II (PPQ-II), the original structure of which was initially referred to the criteria set out in the DSM-IV classification, measures intrusive recollections, avoidance and hyperarousal symptoms (Callahan et al., 2006; DeMier et al., 1996; Szablewska et al., 2025); Instruments demonstrated satisfactory psychometric properties in prior Polish validation studies and were used here as complementary measures of PTSD symptom severity in postpartum period (PPQ-II: Arousal (*α*_C_ = 0.86, *ω*_M_ = 0.87), Avoidance and Intrusion (*α*_C_ = 0.91, *ω*_M_ = 0.91), and the total score (*α*_C_ = 0.92, *ω*_M_ = 0.92) (Szablewska et al., 2025).
- The PTSD-8, a brief screening tool, which comprises eight items that correspond directly to the diagnostic criteria for PTSD as defined in the DSM-5 (American Psychiatric Association, 2022; Mazur et al., 2024). High reliable score (*α*_C_ = 0.89, *ω*_M_ = 0.89) was obtain in Polish version of PTSD-8 questionnaire (Mazur et al., 2024).
  Both instruments were used as screening tools to assess symptom severity and do not constitute a clinical diagnosis of PTSD.

### 2.3 Bias control and data quality assurance

To minimise potential sources of bias:

- Selection bias was reduced by distributing the survey across multiple online communities representing diverse geographic and socioeconomic backgrounds;
- Information bias was limited through the use of validated, standardised self-report instruments and clearly worded items reviewed by experts for comprehension;
- Non-response bias was mitigated by maintaining anonymous participation and allowing flexible completion times;
- Confounding bias related to variations in care models was structurally controlled, as maternity care in Poland is strictly standardised in all public and private hospital departments under the legislative National Standard of Perinatal Care.

Data were screened for logical inconsistencies and extreme outliers by two independent researchers before statistical analyses.

### 2.4 Sample size calculations

The final sample size (*N* = 263) was determined based on the number of complete responses meeting inclusion criteria. To ensure methodological rigor, the adequacy of the final sample size was justified using both an epidemiological approach and a precision-based framework (Lakens, 2022). Based on previously mentioned literature, the prevalence of clinically significant PTSD symptoms after childbirth is estimated at up to 12% (Heyne et al., 2022), with CB-PTSD rates in Poland reaching 6.1% (Murawska et al., 2026). Using Cochran’s sample size formula, assuming an upper-bound prevalence of 12%, a 95% confidence level, and a stringent 4% margin of error, the minimal required sample size to achieve representativeness was 254. Furthermore, evaluating our final collected sample of 263 through the precision-based approach (*N* = (*z* ∙ sd/error)^2^), the dataset guarantees a high degree of accuracy, maintaining the estimation error strictly below 4%. Thus, the sample provides sufficient informative value and estimation precision to reliably investigate the targeted psychological outcomes.

### 2.5 Statistical analysis

The statistical analyses were performed using Jamovi 2.6 and Statistica 13.3. A significance threshold of *α* = 0.05 was applied across all analyses. To address the research questions, General Linear Models were estimated, in which clinical variables served as predictors of post-traumatic stress symptom severity after childbirth. Sociodemographic variables (age, education, place of residence, relationship status), alongside the number of children and the time elapsed between childbirth and study participation, were included as covariates to adjust for their potential confounding effects in alignment with the biopsychosocial framework. Additionally, variables were dichotomised and assessed using odds ratios with corresponding confidence intervals.

## 3 Results

General linear models predicting the severity of post-traumatic stress related to childbirth, as measured by the PPQ-II and PTSD-8, demonstrated acceptable model fit (see Table 2). The models explained 16 and 27% of the variance in PPQ-II and PTSD-8 scores, respectively. Most of the covariates included in the models-namely the number of children, the time elapsed between childbirth and study participation, education level, place of residence and relationship status-were not statistically significant predictors. Maternal age emerged as a significant covariate, but exclusively in the PTSD-8 model. However, this association was extremely weak (*r* ≈ 0.16) and accounted for only 2.5% of the variance in PTSD-8 scores. This indicates that although maternal age may have a marginal effect on symptom reporting on this particular measure, key clinical and obstetric predictors remain robust and largely independent of participants’ sociodemographic background.

**Table 2:** General linear model results of mothers’ probable childbirth-related post-traumatic stress disorders.

Antecedent	Consequent														VIF
	Childbirth-related post-traumatic stress disorder [PPQ-II]							Childbirth-related post-traumatic stress disorder [PTSD-8]							
	*B*	SE	CI 95%		*β*	*t*	*p*	*B*	SE	CI 95%		*β*	*t*	*p*	
			LL	UL						LL	UL				
Constant	37.548	4.624	28.438	46.658	1.405	8.120	< 0.001	22.699	1.911	18.934	26.464	1.311	11.876	< 0.001	
Mode of birth															
Vaginal birth → CS planned	1.093	2.374	−3.584	5.770	0.083	0.460	0.646	0.688	0.981	−1.245	2.622	0.118	0.702	0.484	1.912
Vaginal birth → CS I stage	6.209	2.235	1.806	10.611	0.474	2.778	0.006	2.711	0.924	0.892	4.531	0.466	2.935	0.004	
Vaginal birth → CS II stage	5.487	2.796	−0.022	10.996	0.419	1.962	0.051	2.234	1.156	−0.043	4.511	0.384	1.933	0.054	
Intrapartum complications															
Absence → Occurrence	6.207	2.874	0.544	11.869	0.474	2.159	0.032	2.916	1.188	0.576	5.256	0.501	2.454	0.015	1.151
Near-term complications postpartum haemorrhage															
Absence → Occurrence	2.033	3.104	−4.082	8.149	0.155	0.655	0.513	1.196	1.283	−1.332	3.724	0.206	0.932	0.352	1.175
Near-term complications postpartum curettage															
Absence → Occurrence	8.452	2.946	2.649	14.255	0.645	2.869	0.004	3.732	1.218	1.334	6.131	0.642	3.065	0.002	1.259
Further-term complications															
Absence → Occurrence	3.436	3.707	−3.867	10.738	0.262	0.927	0.355	3.188	1.532	0.170	6.207	0.548	2.081	0.039	1.088
Medicalization															
Absence → Occurrence	−1.672	2.100	−5.808	2.464	−0.128	−0.796	0.427	−0.607	0.868	−2.316	1.103	−0.104	−0.699	0.485	1.412
Prolonged hospital stay															
Absence → Occurrence	−2.319	2.014	−6.285	1.648	−0.177	−1.151	0.251	−0.578	0.832	−2.217	1.062	−0.099	−0.694	0.488	1.358
Facilities during delivery															
Absence → Occurrence	−3.792	2.509	−8.735	1.151	−0.289	−1.511	0.132	−1.892	1.037	−3.935	0.151	−0.325	−1.824	0.069	1.595
Duration of last pregnancy															
Premature → In time	−2.546	2.360	−7.195	2.104	−0.194	−1.079	0.282	−0.547	0.975	−2.469	1.374	−0.094	−0.561	0.575	1.653
Child congenital defect															
Absence → Occurrence	8.776	4.432	0.046	17.506	0.670	1.980	0.049	2.588	1.832	−1.020	6.196	0.445	1.413	0.159	1.053
Neonatal-related hospitalisation															
Absence → Occurrence	4.880	2.091	0.760	8.999	0.372	2.333	0.020	4.094	0.864	2.392	5.797	0.704	4.737	< 0.001	1.895
Postpartum-to-study interval	0.720	0.721	−0.701	2.141	0.062	0.998	0.319	−0.121	0.298	−0.708	0.466	−0.023	−0.406	0.685	1.187
Number of children	0.070	0.970	−1.840	1.980	0.005	0.072	0.942	−0.015	0.401	−0.805	0.774	−0.002	−0.038	0.970	1.235
Education															
Primary → Secondary	−0.261	6.501	−13.067	12.545	−0.020	−0.040	0.968	−1.626	2.687	−6.919	3.667	−0.279	−0.605	0.546	1.341
Primary → Higher	−0.554	6.429	−13.219	12.111	−0.042	−0.086	0.931	−1.094	2.657	−6.329	4.141	−0.188	−0.412	0.681	
Place of residence															
Rural area → City up to 50,000 citizens	−2.705	2.956	−8.527	3.118	−0.206	−0.915	0.361	−1.665	1.222	−4.071	0.741	−0.286	−1.363	0.174	1.221
Rural area → City 50–500,000 citizens	−0.771	2.906	−6.496	4.954	−0.059	−0.265	0.791	−0.899	1.201	−3.265	1.467	−0.155	−0.749	0.455	
Rural area → City over 500,000 citizens	−3.340	2.543	−8.349	1.669	−0.255	−1.313	0.190	−0.737	1.051	−2.808	1.333	−0.127	−0.702	0.484	
Relationship															
Single → Non-formal	−6.781	6.613	−19.809	6.247	−0.517	−1.025	0.306	−0.125	2.733	−5.509	5.260	−0.021	−0.046	0.964	1.306
Single → Formal	−5.014	6.379	−17.579	7.552	−0.383	−0.786	0.433	0.717	2.636	−4.476	5.911	0.123	0.272	0.786	
Age	−0.183	0.209	−0.594	0.228	−0.057	−0.878	0.381	−0.229	0.086	−0.399	−0.059	−0.160	−2.659	0.008	1.305
Model fit	$R_{\mathrm{adj}}^{2}=0.158,$ *F*_(23, 239)_ = 3.135, *p* < 0.001							$R_{\mathrm{adj}}^{2}=0.270,$ *F*_(23, 239)_ = 5.209, *p* < 0.001							

Confidence interval estimate with bootstrap procedure (*N* = 5,000).

*B*, non-standardised coefficient; SE, standard error; CI, confidence interval; LL, lower limit; UL, upper limit; *p*, significance; *t*, Student’s *t*-test results; *β*, standardised coefficient; *R*_adj_^2^, adjusted determination coefficient; *F*, ANOVA fit model results; VIF, variance inflation index.

A bivariate correlation matrix providing an overview of the interrelationships between the main study variables, including sociodemographic and clinical factors, is presented in Supplementary Table S.1.1.

The statistically significant predictors common to both models included caesarean section performed during the 1st stage of labour (*β* = 0.466–0.474), intrapartum complications, related to the need to use obstetric manoeuvres (*β* = 0.474–0.501), and postnatal neonatal-related hospitalisation (*β* = 0.372–0.704). Caesarean section performed during the 2nd stage of labour demonstrated borderline significance in both the PPQ-II model (*p* = 0.051) and the PTSD-8 model (*p* = 0.054). However, methodological literature increasingly emphasizes the importance of evaluating effect sizes and practical relevance rather than relying strictly on arbitrary *α* thresholds (Henson, 2006). Despite falling slightly outside the traditional *p* < 0.05 threshold, the standardised coefficients for 2nd stage caesarean section indicate a moderate effect size that remains clinically meaningful. The magnitude of this effect showed only a minor decrease compared to the 1st stage of labour, which is noteworthy given the conservative nature of a fully adjusted multivariable model. Therefore, we interpret CS during the 2nd stage of labour as a relevant statistical trend with practical implications for predicting postpartum trauma. Planned caesarean section, however, was not a significant predictor. The strongest predictor of PPQ-II and PTSD-8 scores was a short-term labour complication: postpartum curettage (*β* = 0.642–0.645). In the PPQ-II model, the presence of a congenital anomaly in the newborn was also a significant predictor (*β* = 0.670), whereas in the PTSD-8 model, postnatal further-term complications additionally predicted post-traumatic stress (*β* = 0.548).

In the next step, the results were dichotomised based on the specific algorithms established for each instrument. For the PPQ-II (Szablewska et al., 2025), a standard cut-off score of 33 was applied, whereas for the PTSD-8 (Mazur et al., 2024), the criterion was meeting all three core symptom categories. Participants meeting the respective criteria for these tools were classified into the ‘probable PTSD’ group, while those who did not were classified as ‘no probable PTSD’. These dichotomised outcomes were then compared to clinical factors using odds ratios (see Table 3).

**Table 3:** Clinical predictors of probable childbirth-related post-traumatic stress disorder.

Antecedent	Probable childbirth-related post-traumatic stress disorder					
	[PPQ-II]			[PTSD-8]		
	OR	CI 95%		OR	CI 95%	
		LL	UL		LL	UL
Mode of birth						
Vaginal birth → CS planned	1.045	0.411	2.658	1.029	0.444	2.384
Mode of birth						
Vaginal birth → CS I stage	2.659	1.286	5.648	2.883	1.459	5.698
Mode of birth						
Vaginal birth → CS II stage	2.165	0.813	5.768	1.555	0.597	4.052
Intrapartum complications						
Absence → Occurrence	3.107	1.278	7.555	1.756	0.715	4.311
Near-term complications postpartum haemorrhage						
Absence → Occurrence	1.877	0.690	5.111	1.339	0.496	3.613
Near-term complications—postpartum curettage						
Absence → Occurrence	2.331	0.946	5.743	2.026	0.850	4.825
Further-term complications						
Absence → Occurrence	1.794	0.536	6.004	1.891	0.611	5.856
Medicalisation						
Absence → Occurrence	0.606	0.305	1.205	0.653	0.344	1.238
Prolonged hospital stay						
Absence → Occurrence	1.678	0.855	3.294	2.074	1.120	3.841
Facilities during delivery						
Absence → Occurrence	0.278	0.138	0.562	0.333	0.170	0.652
Duration of last pregnancy						
Premature → In time	0.243	0.124	0.476	0.350	0.185	0.660
Child congenital defect						
Absence → Occurrence	5.014	1.403	17.925	1.358	0.336	5.487
Neonatal-related hospitalisation						
Absence → Occurrence	5.165	2.614	10.208	5.095	2.755	9.442

OR, odds ratio; CI, confidence interval; LL, lower limit; UL, upper limit.

Caesarean section performed during the first stage of labour was associated with at least 2.7-fold higher odds of a high post-traumatic stress score. Caesarean section during the second stage of labour was associated with a two-fold increase in the odds of a high PPQ-II score, although this association was less evident for the PTSD-8. Intrapartum complications were linked to a three-fold increase in the odds of a high PPQ-II score. Postpartum curettage was associated with more than a two-fold increase in the odds of high post-traumatic stress related to childbirth. The need for postnatal hospitalisation of the newborn was associated with over five-fold higher odds of high PPQ-II and PTSD-8 scores. Similarly, the presence of a congenital anomaly in the newborn was associated with about five-fold higher odds of elevated post-traumatic stress in the PTSD-8 model.

## 4 Discussion

Our findings confirm that maternal psychological wellbeing in the postpartum period is significantly influenced by the immediate obstetric context. Our analyses revealed that clinical labour and birth factors account for appreciable proportions of postpartum traumatic stress symptoms: explanatory models including mode of birth, intrapartum complications, neonatal-related hospitalisation and congenital anomalies explained approximately 16% of variance in PPQ-II scores and 27% in PTSD-8 scores. Notably, emergency caesarean section in the first and second stages of labour, intrapartum complications, near-term complications like postpartum curettage and neonatal-related hospitalisation emerged as significant predictors, while planned caesarean section did not. Furthermore, the high convergence between the PPQ-II and PTSD-8 assessment tools strengthens the reliability of identifying post-traumatic stress symptoms in this study cohort.

The observed non-significance of planned caesarean section as a predictor merits discussion. While many previous studies have flagged operative delivery in general as a risk factor for postpartum PTSD symptoms, our findings align with recent research showing that it is particularly emergency operative delivery and unresolved intrapartum events that elevate risk (Ginter et al., 2022; Hüner et al., 2024; Orovou et al., 2025). These latter researches emphasise the role of subjective loss of control and unexpected complications rather than the mode of birth itself. The results present in our study substantiate the importance of procedural context: specifically, proactive planning and transparent communication seem to ameliorate the adverse psychological sequelae of caesarean section, whereas the unforeseen nature of unplanned operative delivery maintains a significant predisposition to adverse outcomes. These findings are in alignment with existing literature which emphasizes the protective role of procedural control and adequate preparation in birth experiences (Carter et al., 2022; Hernández-Martínez et al., 2020; Murawska et al., 2026; Silva-Fernandez et al., 2023).

Our data further refine this understanding by demonstrating that caesarean sections performed during the first stage of labour are associated with elevated post-traumatic stress symptoms (PTSD).

This pronounced psychological risk is hypothesized to result from a synergistic effect of multiple traumatic factors inherent to this specific obstetric trajectory, a mechanism substantiated by extant literature (Orovou et al., 2025). The procedure integrates the severe distress associated with an unplanned or emergent intervention with a profound experience of locus of control disruption subsequent to a period of active labour. For the woman, the urgent escalation early in the parturition process often occurs when a vaginal birth remains the dominant expectation, leading to the surgical outcome being subjectively interpreted as a critical failure of the intensely anticipated natural process. This rapid, disorienting transition from the labour ward to an urgent surgical environment fulfils the definitional criteria for a subjectively traumatic event and constitutes a recognised, potent predictor of PTSD (Dekel et al., 2017; Orovou et al., 2025; Xu et al., 2025).

Other studies indicate that instrumental vaginal deliveries (forceps and/or vacuum) may be an equally important predictor of CB-PTSD symptom levels (Carter et al., 2022). As with emergency caesarean sections, the key factor is not only the use of forceps or vacuum extraction, but also the manner of delivery: urgency, complications, communication, respect, support, and the opportunity for shared decision-making (Muraca et al., 2023). Instrumental vaginal deliveries constitute a minority of labours; in Poland, according to the National Health Fund data from 2025 (Narodowy Fundusz Zdrowia, 2026), their percentage was approximately 1.8% of all births; but they are clearly associated with a higher risk of PTSD symptoms than spontaneous deliveries. Unfortunately, the relationship between instrumental deliveries and CB-PTSD symptoms was not investigated in this study.

Conversely, planned caesareans or those performed later in the labour course-following prolonged labour or failed instrumental attempts-may facilitate greater psychological assimilation or situational acceptance, thus providing a partial buffer against severe emotional sequelae (Saulnier et al., 2025; Xu et al., 2025).

These findings underscore that the temporality and procedural context of obstetric intervention-rather than its mere type-are key determinants of childbirth-related trauma risk.

The results of the current study corroborate this principle, thereby accentuating the imperative for sensitive, informed communication and sustained emotional support during emergent clinical decision-making.

Beyond the intrapartum phase, postpartum interventions such as curettage emerged as additional predictors of CB-PTSD in our models. This aligns with previous research indicating that invasive postpartum procedures, particularly those involving unanticipated bleeding or uterine evacuation, can re-trigger the sense of threat and bodily violation that underpins traumatic appraisals of birth (Ayers et al., 2018; Dekel et al., 2017; Garthus-Niegel et al., 2018). These “near-term complications” may carry both physical and symbolic implications-marking an abrupt extension of the birth crisis and prolonging exposure to emergency medical interventions. Perversely, in the study cohort, postpartum haemorrhage was assessed independently and did not emerge as a significant predictor of CB-PTSD symptoms, highlighting the complex, multifactorial nature of postpartum trauma.

The finding that postpartum curettage was the strongest predictor of CB-PTSD symptoms (*β* = 0.645–0.642) warrants closer clinical and psychological interpretation. Postpartum curettage is typically performed for removal of retained products of conception (RPOC) or secondary postpartum haemorrhage. The procedure is frequently urgent, highly painful and usually performed when the woman may already be physically exhausted and emotionally vulnerable. Moreover, despite the widespread use of less invasive methods for RPOC removal, such as hysteroscopy or electric vacuum aspiration (Nir et al., 2022; Perlman and Carusi, 2019), in Polish clinical settings, instrumental postpartum curettage is one of the first-choice interventions according to current recommendations (Rekomendacje Polskiego Towarzystwa Ginekologów i Położników, n.d.). This emergency medical intervention, often performed without anaesthesia and requiring the use of surgical instruments, may be perceived as particularly traumatic by the women in the study group. Unlike a planned procedure, emergency curettage after delivery interrupts the perceived end of labour, creating a sense of unexpected physical violation. Additionally, it abruptly interrupts the long-awaited, early moments of mother-infant bonding, often requiring immediate physical separation from the newborn. Several mechanisms may explain its strong association with CB-PTSD. First, the invasive nature of uterine evacuation, involving instrumental vaginal manipulation, may re-trigger feelings of loss of bodily integrity and loss of control, which are core features of childbirth trauma (Ayers et al., 2016, 2018). Second, the need to perform the procedure under urgent medical circumstances, with limited time for response and informed consent or supportive communication, increases the risk of peritraumatic distress (Dekel et al., 2017). Third, evidence suggests that surgical interventions after childbirth are often perceived as “a betrayal of the expectation that the body is safe after birth” (Ayers et al., 2018). Furthermore, curettage may be perceived by women as a consequence of their own body’s failure or malfunction, which can exacerbate negative appraisals and self-blame, the key factors in the cognitive model of PTSD (Ehlers and Clark, 2000). Clinically, this highlights the need for trauma-informed communication, anticipation, and adequate pain management, even in the acute postpartum period, especially when invasive procedures are necessary.

By contrast, further-term complications that occurred after the immediate postpartum period were less predictive of CB-PTSD severity, suggesting that the temporal proximity and perceived acuteness of medical events during or shortly after childbirth play a decisive role in shaping trauma responses. This pattern can be interpreted within the framework of the Cognitive Model of PTSD (Ehlers and Clark, 2000), which posits that traumatic stress arises from appraisals of persistent threat and poor integration of sensory-emotional memories. Invasive obstetric procedures-particularly those involving unanticipated bleeding or uterine manipulation-may heighten perceptions of bodily violation and loss of control, reinforcing maladaptive cognitive processing. Similar mechanisms are described by the Dual Representation Theory (Brewin et al., 1996; Brewin and Burgess, 2014), where trauma is encoded predominantly in sensory memory systems, leading to intrusive re-experiencing. Thus, postpartum medical interventions may act as stressors not only physiologically but also cognitively, by disrupting women’s perceived agency and continuity of the birthing experience.

Neonatal health variables also demonstrated strong and consistent associations with maternal post-traumatic stress. In particular, neonatal-related hospitalisation increased the odds of postpartum post-traumatic stress symptom severity nearly five-fold, while congenital anomalies were predictive of higher PPQ-II scores. These findings echo prior meta-analyses identifying neonatal complications, mother-infant separation and NICU admission as key determinants of maternal distress (Di Chiara et al., 2022; Malouf et al., 2024; Orovou et al., 2023). Separation immediately following birth may disrupt the early bonding process and amplify a mother’s sense of helplessness, thereby reinforcing intrusive and avoidance symptoms typical of CB-PTSD. Importantly, these factors reflect a dyadic stress response, extending the impact of birth trauma beyond the mother to the mother-infant relationship and family adjustment. Previous predictive models have identified four significant variables placing women with a history of traumatic childbirth at risk for the development of CB-PTSD. These variables encompass insufficient social support, poor coping mechanisms, the subjective experience of “threatened death,” and the perceived or actual trauma associated with “injury to the baby” (Saulnier et al., 2025).

The interplay between obstetric emergencies, peripartum interventions and compromised neonatal outcomes underscores that CB-PTSD risk frequently emerges from a cascade of acute clinical stressors rather than resulting from a single traumatic event. This aligns robustly with multidimensional conceptualisations of perinatal trauma, which integrate biological, cognitive and relational determinants (Dekel et al., 2017; Horsch et al., 2024; Qureshi et al., 2025; Sentilhes et al., 2017). The coexistence and chronological proximity of multiple stressors within a short timeframe—such as an unplanned, urgent caesarean section immediately followed by complex postpartum procedures or extended neonatal hospitalisation—is hypothesized to produce a significant cumulative psychological load. This sequential trauma challenges the mother’s cognitive and emotional resources, often exceeding her capacity for adaptive coping and processing (Dekel et al., 2017; Xu et al., 2025).

Furthermore, this rapid succession of high-intensity events can inhibit effective emotional recovery and meaning-making, preventing the necessary pause required for integrating the traumatic experience. The ongoing stress associated with the infant’s health (e.g., NICU admission) acts as a powerful secondary stressor, maintaining the mother in a state of hyperarousal and chronic vigilance, which significantly potentiates the development and persistence of PTSD symptoms (Modarres et al., 2012; Orovou et al., 2025). This constant activation, stemming from both the immediate procedure and the subsequent clinical reality, directly increases the likelihood of the memory being encoded as a traumatic event (Modarres et al., 2012).

Although clinical interventions are critical for birth-related PTSD, it is equally important to acknowledge the broader scope of trauma that women bring into the maternity care setting. The perinatal experience frequently intersects with a woman’s pre-existing psychological history. Prior adversities, such as Adverse Childhood Experiences (ACEs), intimate partner violence, miscarriages, stillbirths or abortion, can significantly compound vulnerability to perinatal psychological distress (Giannandrea et al., 2013; Mackle et al., 2023). Furthermore, routine obstetric care can worsen psychological distress due to both the nature of the obstetric exam and the loss of bodily autonomy inadvertently re-triggering these past traumas (Horsch et al., 2024).

Thus, there is a clear need for the implementation of Trauma-Informed Care (TIC) in maternity care settings. The proposed rules of conduct are based on the 5 “R” Principles of Trauma-Informed Care: Realize, Recognize, Respond, Resist Re-traumatization, and Resilience (Schroeder et al., 2025). Practically, this means healthcare providers must realize the influence of previous life experiences and learn how to recognize subtle signs of traumatic stress during routine obstetric procedures. Furthermore, the response of the clinicians should be based on empathy, actively resist re-traumatization by prioritizing bodily autonomy and continuous consent, and ultimately foster patient resilience through collaborative and empowering care. This paradigm shift in care will move healthcare providers beyond simply reacting to acute birth trauma toward a proactive approach that universally recognizes, respects, and appropriately responds to the cumulative trauma burden many women carry.

From a broader perspective, the current findings contribute to understanding CB-PTSD within health systems characterised by high medicalisation of childbirth. Poland, similar to several Central and Eastern European countries, continues to report among the highest caesarean section rates on the continent (Amyx et al., 2024). However, similar challenges are observed globally: from rapidly rising caesarean section rates in Latin America and parts of Asia to the coexistence of underuse and overuse of surgical interventions in low- and middle-income countries (Betrán et al., 2016; Betran et al., 2021; Boerma et al., 2018). While high intervention rates reflect improved obstetric safety, they also highlight the need for balanced, trauma-informed perinatal care addressing both physical and psychological outcomes across all healthcare systems, not just in Europe (Ayers et al., 2024; Berring et al., 2024; Heyne et al., 2022).

Additionally, the incidence of CB-PTSD tends to be higher in the Middle East and some low- and middle-income regions (LMIC), which is related to limited access to healthcare and social inequalities (Jenkins et al., 2024). These findings align with recent observations in the Polish population (Burdecka and Szablewska, 2025), in which it was demonstrated that postpartum mental health of women is significantly influenced by a combination of obstetric and sociodemographic factors, like younger maternal age, informal relationship status, or financial hardship. Furthermore, living in rural areas and experiencing a non-physiological delivery were notably associated with a higher risk of developing PTSD symptoms after childbirth (Burdecka and Szablewska, 2025). Consequently, this indicates the critical need for identifying factors predisposing women to CB-PTSD and implementation of targeted screening among highly vulnerable groups, such as women facing economic problems or recovering from traumatic childbirths.

At the same time, the pattern of findings observed in the present study suggests that these clinical variables may not represent entirely independent sources of risk. Rather, emergency caesarean delivery, intrapartum complications, postpartum curettage, neonatal-related hospitalisation and congenital anomalies appear to share a common characteristic: they constitute acute, clinically significant stressors occurring within a relatively short period surrounding childbirth. Viewed from this perspective, the present results may reflect not only the impact of specific medical events but also a broader burden of acute obstetric stress exposure.

Although the present study was not designed to test a cumulative-risk model, future research may examine whether the accumulation of acute obstetric and neonatal complications contributes to childbirth-related PTSD symptoms beyond the effects of individual clinical factors.

Such an interpretation is broadly consistent with established theories of post-traumatic stress, including the cognitive model of PTSD (Ehlers and Clark, 2000), which emphasizes the role of threat appraisals and trauma processing, and Janoff-Bulman’s (1992) assumptive world, which highlights the disruption of beliefs regarding safety, predictability, and personal agency following highly stressful events.

Overall, the present findings expand current knowledge by demonstrating that acute clinical factors-spanning labour, immediate postpartum interventions and neonatal complications-collectively shape postpartum post-traumatic stress responses. These results support the integration of trauma-informed approaches in perinatal healthcare policies and underline the importance of interdisciplinary collaboration between obstetric, midwifery and psychological services to promote maternal and infant wellbeing.

### 4.1 Strengths of the study

This study extends previous work on childbirth-related post-traumatic stress by focusing on a comprehensive range of maternal and neonatal clinical predictors within a large, community-based sample. By integrating obstetric, postpartum and infant health variables into the same analytical models, a multidimensional view is offered on how medical and situational factors jointly shape postpartum trauma risk.

A particular strength lies in the simultaneous use of two validated measures of post-traumatic stress (PPQ-II and PTSD-8), which increases the robustness of findings across different conceptualisations of trauma symptomatology. The inclusion of detailed obstetric and neonatal variables, such as stage-specific caesarean sections, postpartum interventions and infant hospitalisation, provides clinically actionable insights, rarely addressed together in prior research.

Furthermore, the study contributes novel data from a Central and Eastern European setting-a region underrepresented in perinatal mental health research-offering valuable contextual evidence for international comparisons. By drawing on a national sample and adhering to rigorous statistical procedures, the study allows to enhance the understanding of perinatal trauma mechanisms in healthcare systems with high medicalisation of childbirth.

### 4.2 Limitations

Several limitations should be acknowledged when interpreting these findings. Firstly, the cross-sectional design precludes conclusions regarding causality or temporal ordering between clinical events and post-traumatic stress symptoms. Longitudinal follow-up studies are warranted to determine whether the identified factors predict the persistence or remission of symptoms over time.

Second of all, the use of an online, self-selected sample introduces potential sampling bias. Although the sample size was adequate for statistical power, participation was voluntary and limited to individuals with internet access, potentially underrepresenting socially or clinically marginalised groups.

Next, all measures relied on self-reporting, which may be affected by recall or response biases. The absence of clinical diagnostic interviews limits the ability to establish formal PTSD diagnoses according to DSM-5 or ICD-11 criteria.

Fourth, while the inclusion of a broad set of clinical variables strengthens ecological validity, the lack of certain psychosocial measures (e.g., perceived support, prior trauma history) restricts interpretation of how medical and psychological factors interact.

It should be also noted that due to the self-reported nature of the data and the lack of access to official medical records, our study could not definitively link specific medical interventions (e.g., postpartum curettage) to their exact clinical indications on a case-by-case basis. To address this, clinical events and procedures were measured as distinct variables.

A significant limitation of this study is the potential for selection bias due to informative missingness. Since the data were collected via Google Forms, which does not record incomplete attempts, the exact dropout rate and the characteristics of respondents who dropped out the survey remain unknown. It is highly likely that women experiencing severe postpartum trauma or psychological distress found the questions provocative and withdrew from the study before it was reported. Therefore, our findings regarding the prevalence and severity of CB-PTSD symptoms might be underestimated, and the sample may overrepresent individuals with better psychological coping mechanisms.

Furthermore, the proposed AOS framework is a conception, designed to generate hypotheses; therefore, it requires future prospective verification, preferably using formal mediation analyses to confirm the proposed psychological mechanisms.

Additionally, the exclusion of forceps and vacuum extraction deliveries is a notable limitation of the present study. Operative vaginal births are often associated with physical and psychological stressors and their exclusion restricts our ability to fully evaluate the spectrum of expedited birth modes and their specific influence on CB-PTSD symptoms. Future research should specifically include this variable to capture the context of instrumental birth-related psychological outcomes.

Finally, due to data collection occurring within a single national context and during a short recruitment window, the generalisability to other healthcare systems or cultural settings should be made with caution. In future studies, mixed-methods or prospective designs should be adopted, integrating partner perspectives and including clinician-assessed outcomes to deepen understanding concerning the multifactorial nature of postpartum post-traumatic stress.

## 5 Conclusion

In this national cross-sectional study, emergency caesarean sections, intrapartum complications and neonatal hospitalisation were the strongest predictors of childbirth-related post-traumatic stress symptoms, accounting for up to one-quarter of the observed variance.

These findings indicate that the burden of childbirth-related trauma-recognised as a major underlying cause of overall maternal postpartum distress in highly medicalised maternity systems is closely tied to the intensity and temporal proximity of obstetric and neonatal stressors. Consequently, these results strongly suggest the need to broaden perinatal mental health surveillance, moving beyond conventional psychosocial screening to incorporate context-specific clinical risk factors.

Implementing structured, trauma-informed postpartum pathways-including early psychological assessment following emergency interventions-could strengthen maternal mental health outcomes and align national perinatal care with contemporary European standards. Specifically, this approach requires moving beyond general psychosocial screening to deploy targeted psychological follow-ups for women who have undergone emergency caesarean sections, invasive postpartum procedures like curettage, or early separation due to neonatal hospitalisation. Furthermore, integrating routine practices that guarantee continuous consent, clear communication, and robust pain management during acute interventions will actively resist re-traumatization and foster better emotional recovery.

## Data Availability

The original contributions presented in the study are included in the article/Supplementary material, further inquiries can be directed to the corresponding author.
