# Supplementary material for: Maternal and perinatal factors associated with childbirth-related post-traumatic stress symptoms: a cross-sectional study
Source: Front Psychol. 2026 Jul 13;17:1779037. doi: 10.3389/fpsyg.2026.1779037 (PMC13402402; doi:10.3389/fpsyg.2026.1779037)
Supplement: Supplementary file 1 [file Data_Sheet_1.ZIP › Sup. material.docx]

**Appendix**

**S1 Appendix Bivariate correlation matrix for the main study variables and CB-PTSD**

**Table S1.1.**

*Bivariate correlations between all tested variables and mothers’ probable childbirth-related post-traumatic stress disorders*

| Tested variables | Childbirth-related Post Traumatic Stress Disorder | | | | | | | | | | |
| --- | --- | --- | --- | --- | --- | --- | --- | --- | --- | --- | --- |
|  | [PPQ-II] | | | | |  | [PTSD-8] | | | | |
|  | *r_pbi_* | *r* | 95 *CI* | | *p* |  | *r_pbi_* | *r* | 95 *CI* | | *p* |
|  |  |  | *LL* | *UL* |  |  |  |  | *LL* | *UL* |  |
| Mode of birth |  |  |  |  |  |  |  |  |  |  |  |
| Vaginal birth → CS planned | 0.024 |  | -0.111 | 0.167 | 0.747 |  | 0.033 |  | -0.094 | 0.168 | 0.658 |
| Vaginal birth → CS I stage | 0.234 |  | 0.092 | 0.374 | 0.001 |  | 0.245 |  | 0.108 | 0.385 | 0.001 |
| Vaginal birth → CS II stage | 0.155 |  | 0.003 | 0.315 | 0.049 |  | 0.153 |  | 0.011 | 0.300 | 0.053 |
| Intrapartum complications  Absence → Occurrence | 0.171 |  | 0.029 | 0.304 | 0.006 |  | 0.176 |  | 0.033 | 0.315 | 0.004 |
| Near-term complications Postpartum hemorrhage  Absence → Occurrence | 0.086 |  | -0.063 | 0.234 | 0.165 |  | 0.098 |  | -0.047 | 0.239 | 0.114 |
| Near-term complications Postpartum curettage  Absence → Occurrence | 0.170 |  | 0.039 | 0.299 | 0.006 |  | 0.175 |  | 0.023 | 0.319 | 0.005 |
| Further-term complications  Absence → Occurrence | 0.063 |  | -0.062 | 0.199 | 0.306 |  | 0.132 |  | -0.013 | 0.272 | 0.032 |
| Medicalization  Absence → Occurrence | -0.108 |  | -0.233 | 0.017 | 0.080 |  | -0.092 |  | -0.214 | 0.031 | 0.139 |
| Prolonged hospital stay  Absence → Occurrence | 0.087 |  | -0.034 | 0.208 | 0.162 |  | 0.187 |  | 0.070 | 0.307 | 0.002 |
| Facilities during delivery  Absence → Occurrence | -0.265 |  | -0.383 | -0.141 | <0.001 |  | -0.291 |  | -0.402 | -0.174 | <0.001 |
| Duration of last pregnancy  Premature → In time | -0.248 |  | -0.372 | -0.118 | <0.001 |  | -0.292 |  | -0.410 | -0.169 | <0.001 |
| Child congenital defect  Absence → Occurrence | 0.129 |  | 0.010 | 0.231 | 0.037 |  | 0.093 |  | -0.014 | 0.188 | 0.132 |
| Neonatal-related hospitalization  Absence → Occurrence | 0.273 |  | 0.157 | 0.389 | <0.001 |  | 0.406 |  | 0.295 | 0.513 | <0.001 |
| Postpartum-to-study interval |  | 0.116 | <0.001 | 0.235 | 0.060 |  |  | 0.026 | -0.100 | 0.149 | 0.678 |
| Number of children |  | 0.020 | -0.102 | 0.141 | 0.752 |  |  | -0.023 | -0.139 | 0.099 | 0.709 |
| Education |  |  |  |  |  |  |  |  |  |  |  |
| Primary → Secondary | -0.039 |  | -0.356 | 0.245 | 0.782 |  | -0.102 |  | -0.425 | 0.206 | 0.461 |
| Primary → Higher | -0.023 |  | -0.181 | 0.110 | 0.741 |  | -0.037 |  | -0.196 | 0.100 | 0.592 |
| Place of residence |  |  |  |  |  |  |  |  |  |  |  |
| Rural area→ City up to 50 000 citizens | -0.173 |  | -0.384 | 0.063 | 0.144 |  | -0.270 |  | -0.478 | **-0.036** | **0.021** |
| Rural area→ City 50-500 000 citizens | -0.047 |  | -0.269 | 0.179 | 0.674 |  | -0.137 |  | -0.353 | 0.092 | 0.218 |
| Rural area→ City over 500 000 citizens | -0.133 |  | -0.303 | 0.045 | 0.086 |  | -0.131 |  | -0.297 | 0.044 | 0.090 |
| Relationship |  |  |  |  |  |  |  |  |  |  |  |
| Single → Non-formal | -0.087 |  | -0.375 | 0.134 | 0.528 |  | 0.017 |  | -0.158 | 0.221 | 0.903 |
| Single → Formal | -0.046 |  | -0.197 | 0.060 | 0.503 |  | 0.015 |  | -0.060 | 0.116 | 0.830 |
| Age |  | -0.022 | -0.136 | 0.092 | 0.718 |  |  | -0.105 | -0.222 | 0.013 | 0.090 |

*Note.* Confidence interval estimate with bootstrap procedure (*N* = 5000).

*r* – Pearson coefficient, *r_pbi_* – point-biserial coefficient, *CI* – confidence interval, *LL* – lower limit, *UL* – upper limit, *p* – significance
